# Behavioral Indicators on a Mobile Sensing Platform Predict Clinically Validated Psychiatric Symptoms of Mood and Anxiety Disorders

**DOI:** 10.2196/jmir.6678

**Published:** 2017-03-16

**Authors:** Skyler Place, Danielle Blanch-Hartigan, Channah Rubin, Cristina Gorrostieta, Caroline Mead, John Kane, Brian P Marx, Joshua Feast, Thilo Deckersbach, Alex “Sandy” Pentland, Andrew Nierenberg, Ali Azarbayejani

**Affiliations:** ^1^ Cogito Corporation Boston, MA United States; ^2^ Department of Natural and Applied Sciences Bentley University Waltham, MA United States; ^3^ VA Boston Healthcare System National Center for Posttraumatic Stress Disorder Boston, MA United States; ^4^ Boston University School of Medicine Boston, MA United States; ^5^ Massachusetts General Hospital, Harvard Medical School Bipolar Clinic and Research Program, Department of Psychiatry Boston, MA United States; ^6^ Massachusetts Institute of Technology Media Lab Cambridge, MA United States

**Keywords:** mHealth, post-traumatic stress disorders, depression, behavioral symptoms

## Abstract

**Background:**

There is a critical need for real-time tracking of behavioral indicators of mental disorders. Mobile sensing platforms that objectively and noninvasively collect, store, and analyze behavioral indicators have not yet been clinically validated or scalable.

**Objective:**

The aim of our study was to report on models of clinical symptoms for post-traumatic stress disorder (PTSD) and depression derived from a scalable mobile sensing platform.

**Methods:**

A total of 73 participants (67% [49/73] male, 48% [35/73] non-Hispanic white, 33% [24/73] veteran status) who reported at least one symptom of PTSD or depression completed a 12-week field trial. Behavioral indicators were collected through the noninvasive mobile sensing platform on participants’ mobile phones. Clinical symptoms were measured through validated clinical interviews with a licensed clinical social worker. A combination hypothesis and data-driven approach was used to derive key features for modeling symptoms, including the sum of outgoing calls, count of unique numbers texted, absolute distance traveled, dynamic variation of the voice, speaking rate, and voice quality. Participants also reported ease of use and data sharing concerns.

**Results:**

Behavioral indicators predicted clinically assessed symptoms of depression and PTSD (cross-validated area under the curve [AUC] for depressed mood=.74, fatigue=.56, interest in activities=.75, and social connectedness=.83). Participants reported comfort sharing individual data with physicians (Mean 3.08, SD 1.22), mental health providers (Mean 3.25, SD 1.39), and medical researchers (Mean 3.03, SD 1.36).

**Conclusions:**

Behavioral indicators passively collected through a mobile sensing platform predicted symptoms of depression and PTSD. The use of mobile sensing platforms can provide clinically validated behavioral indicators in real time; however, further validation of these models and this platform in large clinical samples is needed.

## Introduction

Exploring behavioral patterns has tremendous potential to aid clinicians and patients in the real-time recognition and treatment of symptoms and symptom clusters across a variety of disease states. Early recognition of subsyndromal mood and anxiety symptoms is crucial to reduce the pernicious impact of chronic psychological distress and loss of function [1-3]. Yet many affected individuals are unable to receive timely and adequate mental health resources [4]. A key objective of the National Institute of Mental Health’s strategic plan is to identify clinically useful behavioral indicators to determine effective intervention strategies and deliver those interventions at the appropriate time in the illness trajectory [5].

Barriers exist to objectively identifying behaviors in real time. Mobile sensing platforms allow for not only the collection of these behavioral indicators, but also can provide the complex architecture for securely storing, analyzing, and providing feedback. More specifically, mobile sensing platforms can enable the identification and tracking of behaviors (eg, mood, fatigue, social connectedness, physical isolation) from digital trace data passively collected from sensors embedded on mobile devices (eg, call logs, global positioning system [GPS] meta-data, phone activity) [6]. However, this approach still needs to be clinically validated and shown to be scalable; the application of mobile sensing platforms in clinical care is currently limited. A National Institutes of Health (NIH) mHealth evidence workshop cautioned that, despite the promise of these new technologies in clinical care, theoretically based empirical evidence is needed [7]. In this paper, we present a field trial of the implementation of a mobile sensing platform to provide clinically validated behavioral indicators of symptoms of depression and PTSD.

Digital trace data has been used to track and predict behavioral outcomes (eg, GPS data to predict friendship networks amongst graduate students [8] and location and proximity data to predict changes in an individual’s physical health [9]). Mobile sensing platforms using mobile phone trace data have shown some success in predicting self-reported depression [10,11]; however, they have focused on diagnosis and not on tracking specific symptoms.

In addition to digital trace data, the analysis of cues from the voice as a behavioral indicator is another tremendous potential of mobile sensing platforms. Vocal cues can provide valuable insights into physical and mental states, not just through lexical content but also through prosody, voice quality, and overall tone of voice. Analysis of vocal cues can be useful for inferring emotional states [12,13]; for example, various aspects of vocal prosody have been linked to depression severity [14-16]. Vocal cue analysis has recently been supported in a clinical use-case for detecting and telemonitoring symptom progression in Parkinson’s disease [17-19]. However, the potential of audio processing has been largely understudied for clinical use cases outside of Parkinson’s disease. Human experts can identify changes in speech properties of depressed individuals [20] and recent efforts have been made to quantify these vocal features [21].

There is potential to complement or replace traditional methods of assessing mental health and well-being that rely upon an individual’s self-report (ie, surveys, questionnaires, diagnostic interviews) with passively collected indicators derived from behavioral indicators and vocal cues [22,23]. Clinicians would not have to rely solely on subjective retrospective self-reports of symptoms during clinical visits that are subject to recall and other response biases or symptom tracking by self-report that has low levels of sustained adherence.

Identifying and tracking behavioral indicators also has clinical relevance beyond diagnosis. These behavioral indicators, even if validated against a specific clinical symptom, can provide information to clinicians across a number of chronic or comorbid conditions. Patients can have complex profiles of symptoms and underlying behavioral patterns that can be present in a number of different conditions. The Research Domain Criteria (RDoC) framework emphasizes the clinical value for considering underlying behaviors and thinking across categories of mental health disorders [24]. Having access to objectively measured behavioral indicators can help subtype mood and anxiety disorders and recognize patterns in individual patients.

The goal of this project was to test the ability of a scalable mobile sensing platform to collect, store, and analyze objective behavioral indicators underlying mood and anxiety disorder symptoms. Specifically, we hypothesized that models of behavioral indicators of PTSD and depression symptoms could be derived from mobile-based digital trace data and auditory signals.

## Methods

### Participants

Participants were recruited through Web-based advertisements and local veteran service organizations. Inclusion criteria included: age 18 years or older, English speaker, current subscriber to a cellular and data plan, and if they reported at least one symptom of PTSD or depression (measured by Primary Care PTSD Screen, PC-PTSD [25] and the Patient Health Questionnaire, PHQ-2 [26]). Participants were excluded if they had plans to permanently leave the Boston area during the study period, if they shared their cell phone with another person, or if they were active duty military personnel. The protocol was approved by an accredited institutional review board, and all participants were consented. Additional participant and safety details are included in the Multimedia Appendix 1.

### Procedure

Each participant had an initial study visit, 12 weeks of data gathering, and a follow-up study visit. At the initial visit, participants completed a baseline questionnaire and replaced their existing phone with the study mobile phone, equipped with the research app. At the time of the study, the app was limited to a small number of devices so participants transferred their cellular plan and all personal cell phone data to the study Android device. The mobile app has since been made compatible across devices.

The participant used the phone as usual during the study period. In addition, participants were instructed to leave an audio diary entry in the app at least weekly. The audio recordings were designed to be short “voicemail” style entries about how participants were feeling or how their days were going. At the end of the 12-week period, participants returned to the study site and completed a semistructured clinical interview with a trained clinician. In addition, participants completed a close-out survey, were debriefed, and the mobile app and all study data were removed from the phone. Participants were paid US $15/hour for study visits, a 1-time US $50 incentive for completing at least 75% of all assessments, and given the option of keeping the study phone (valued at US $300).

### Mobile Sensing Platform Architecture

Digital trace data (Table 1) were collected on intermittent fixed schedules from predefined, configured probes built into the phone’s operating system. The mobile software data gathering and analysis platform was developed by Cogito with funding from the Defense Advance Research Project Agency (DARPA). Details of the platform architecture are in the Multimedia Appendix 1.

**Table 1:** Categories of digital trace data.

Category	Description
Activity	The physical handling of the phone. The phone hardware includes an accelerometer and gyroscope. The gyroscope was used to determine angle, velocity, direction, and acceleration of the phone. The accelerometer provided data on rate of rotation on the X, Y, and Z axes.
Social	How the user is interacting with others through the phone. Collected the time and deidentified descriptor (see privacy and security section) of all outgoing and ingoing phone calls and SMS (short message service, SMS) text messages.
Location	Where the phone is physically located. Data are combined by the phone operating system from global positioning service (GPS), mobile phone tower triangulation, and WiFi network locations. These data consist of time stamp, longitude, and latitude readings. WiFi network names, WiFi access, usernames, or passwords were not collected.
Device interaction	When the phone is being used. Data are timestamps of when the phone screen is turned on or activated and when it is turned off. We did not record app usage, keystrokes, or any other measure of active use of the device.
Device information	Data describing the physical device. This included variables on phone make, model, battery status (% full), and phone operating system version. This data is used for quality assurance testing.
Vocal cues	Digital recordings of audio-diaries were processed to extract measurements related to speaking, rate, prosody, intonation, and voice quality. These measurements were computed on short-term overlapping frames and then aggregated (using descriptive statistics) over the entire audio diary entry. The lexical content of the recordings was not analyzed.

#### Data Privacy and Security

Participants’ data were protected by a NIH Certificate of Confidentiality, and utilized best practice technical approaches to protect identity. This included both the identity of the participant and the identity of individuals in contact, and communication log data. Encrypted, hashed, deidentified descriptors were used to label all data. Details on the security methodology and algorithms are in the Multimedia Appendix 1.

### Measures

#### Main Outcomes

Clinical symptoms were measured through validated clinical interviews with a trained clinician using the depression and PTSD modules of the Structured Clinical Interview for Mental Disorders (SCID) [27]. We focused our models on 4 behaviorally relevant symptoms (Depression A1—depressed mood most of the day; Depression A2—diminished interest or pleasure in all or most activities; Depression A6—fatigue or loss of energy; and PTSD C2—avoids activities, places, people).

#### Digital Trace Data

Digital trace data collected on the mobile sensing platform fell into 6 categories (Table 1). Details on the schedule and quantity of digital trace data gathered are in the Multimedia Appendix 1.

#### Additional Measures

Participants reported age, race, current annual income, employment, level of education, marital status, and veteran status. At the end of the study participants also reported ease of use, interest in future use, and their perception of data sharing and privacy concerns for both personal and anonymized health data. All questions were 1 (very unlikely) to 5 (very likely) Likert questions. Survey instruments are in Multimedia Appendix 1.

### Analysis

The primary analysis goal was to develop models utilizing digital trace data and audio recordings to predict the presence of clinician-assessed psychiatric symptoms. A secondary goal was to have a model whose inputs reflected types and quantities of data that could be realistically gathered in real world clinical environments with minimal participant burden. A third goal to facilitate clinical utilization and acceptability by clinicians was to develop models with a hypothesis-driven approach from data sources which were clinically interpretable.

Three symptoms of depression and PTSD with strong behavioral components were chosen a priori as target goals for model development from digital trace data (fatigue, interest in activities, and social connectedness). One symptom of depression (depressed mood) was chosen a priori as the target goal for model development from audio data. For each symptom, a binary target variable was defined to characterize the presence or absence of the symptom. To best model clinically relevant differences in symptomatology, we removed participants with only subthreshold symptoms or with insufficient information to determine symptom presence or absence.

Participant audio and mobile features were derived using the most recent week of data (1 week of data prior to the SCID symptom assessment). An initial modeling approach predicted symptoms based on the entire 12-week data collection period; however, high discrimination of the classes was achieved using features temporally closer to the target assessment.

For the 3 models trained from digital trace data, we used the following methodology. Through data exploration and a priori hypotheses on how mobile phone data would capture relevant behaviors, we created a set of features using descriptive statistics over a week of digital trace data. These features included means, counts, and standard deviations of social and location digital trace data (number of texts received in a week, number of minutes spent on outbound calls in the last week). Activity data (see Table 1) was excluded after extensive data exploration failed to yield conceptually meaningful features. Device interaction data (see Table 1) was excluded as data exploration determined that events were being captured for push notifications, not just user-device interactions; therefore, the interaction data did not accurately represent user behavior. The set of features were further reduced by cluster analysis to enhance interpretability on the resulting models and improve prediction performance. The set of 14 features were reduced to prevent over-fitting and interfeature correlation. Reduction method consisted of identifying clusters of features such that within group correlation is high with respect to between group correlation, which highlighted 3 clusters that conceptually matched a digital trace data source (location, social calls, social texts). From each grouping, a single feature was chosen, that conceptually matched a digital trace data source (location, social calls, social texts). Combinations of features were utilized to create 10 candidate models per symptom. Candidate models consisted of logistic models describing the probability of having the corresponding symptom. To select the best model from the list of candidates, we utilized 10-fold cross validation. The model with the highest cross-validated area under the ROC curve (AUC) was chosen (Table 2).

**Table 2:** Model characteristics and performance.

Symptom target	Input features	Cross-validated area under the curve (AUC)
Depressed mood most of the day	MeanPitchVar+MeanVocalEffort+MeanVocalEffort:MeanPitchVar	.74
Diminished interest or pleasure in all or most activities	sms.address.count+travel.distance.sum	.56
Fatigue or loss of energy	call.out.sum+sms.address.count	.75
Avoid activities, places, people	call.out.sum+sms.address.count+(call.out.sum)(sms.address.count)	.83

One model was trained using audio data. The audio was sampled at 8 kHz with 16-bit precision. M4A compression was applied to audio captured by the device and the format was subsequently converted to waveform audio file format (WAV) before computing audio features. Candidate audio features to develop this model were defined by an existing library of features providing temporal information about voice quality, prosody, and intonation. Audio features were aggregated over time as an average and standard deviation per recording. A penalized logistic regression approach, least absolute shrinkage and selection operator (LASSO) [28], was used to reduce the initial set of features using the depressed mood symptom target as the dependent variable. The penalization parameter was determined by 10-fold cross validation. Five candidate features remained following this reduction strategy. Candidate models were created in the same fashion as models created from digital trace data, and the model with the highest cross-validated AUC following 10-fold cross-validation was selected.

Details on the set of reduced features for each model, representative examples from the published literature which include audio feature algorithms, and the full list of candidate models are in the Multimedia Appendix 1.

## Results

### Participant Characteristics

Ninety-five participants consented to the study. One participant called to withdraw and 21 were withdrawn due to technical issues (most commonly a lost phone). Seventy-three participants (67% male, 48% non-Hispanic white, 33% veterans) completed all 12-weeks of data collection and a follow-up clinical interview (see Multimedia Appendix 1 for detailed participant characteristics). There was no difference between participants who completed the study and those that did not in terms of demographic characteristics. All participants reported a lifetime history of at least one traumatic event during clinical interview. The 12-week dataset included 1217 participant audio recordings and 51,080,131 digital trace data points.

### Models and Model Fit

**Figure 1:**
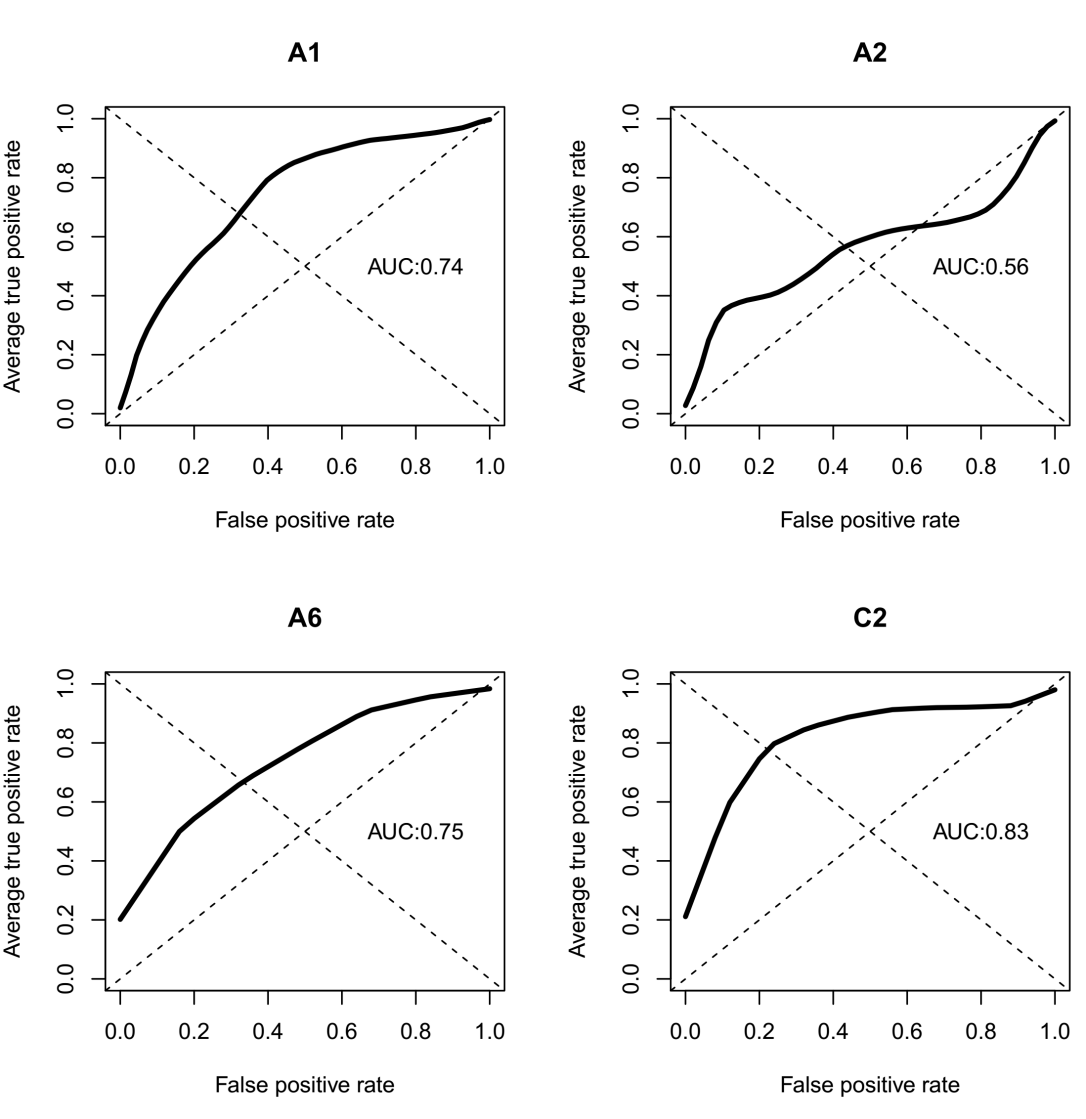
Receiver operating characteristic curves (ROC) and area under the curve (AUC).

The resulting models for each of the 4 dependent variable modeling targets are in Table 2. See Figure 1 for receiver operating characteristic (ROC) curves. Detailed model performance, including positive and negative predictive values and accuracy at varying thresholds, are included in the Multimedia Appendix 1. At a .5 threshold, models predicted clinically assessed symptoms of depression and PTSD (cross-validated AUCs for depressed mood .74, fatigue .56, interest in activities .75, and social connectedness .83). No information on prior diagnoses, current comorbidities, lifestyle, or demographic characteristics was included in the models to achieve these AUCs.

### Acceptability and Feasibility

Adherence to study protocol was extremely high with 96% of participants (N=70) completing at least one audio diary per week. Participants found the mobile app easy to use (Mean 4.05, SD 1.14) and were interested in using the app in the future (Mean 3.59, SD 1.27). Overall participants reported moderate comfort with sharing individual data (Mean 2.48, SD 0.91) and anonymized data (Mean 3.00, SD 1.23). There were no significant differences by age, gender, or veteran status in comfort with sharing. For individualized data, participants were most comfortable with sharing their personal data with primary care physicians (Mean 3.08, SD 1.22), mental health providers (Mean 3.25, SD 1.39), and medical researchers (Mean 3.03, SD 1.36). Participants reported the least comfort with sharing individualized data with insurance providers (Mean 1.63, SD 1.06) and friends (Mean 1.95, SD 1.12).

## Discussion

### Principal Findings

In this study, we examined the use of a mobile sensing platform to passively collect and analyze digital trace and voice data as behavioral indicators of clinically-validated symptoms of depression and PTSD. The models were predictive of clinician-assessed symptoms of depressed mood, fatigue, interest in activities, and social connectedness. This study highlights the power of mobile sensing platforms to gather and compute behavioral indicators of symptoms of mood and anxiety disorders.

To have clinical relevance, modeling strategies for behavioral indicators should be parsimonious (simple), objective (unbiased), and actionable (useful in real-world clinical settings). The modeling strategy in the present analysis was parsimonious, distilling millions of raw data points into a small number of key features. The models were objective, utilizing passively collected digital trace data, and extracted vocal features of tonality and speaking style. Adding self-reported predictors to the models reported here did not significantly increase model performance. There are established biases in self-reporting of mental states [29]. Biases may be intentional or unintentional, as the disorders themselves can change the way patients perceive their own actions. Finally, the models were actionable. Predictive models that have been developed for other disorders, including suicidality, rely on social determinants and clinical characteristics that have to be extracted from electronic medical records [30]. Clinicians may want to know what is happening with patients, but asking patients may not always yield accurate results [31,32].

In addition, these behavioral indicators described by the models are clinically meaningful. The models are based off of clinician-assessed symptoms and not self-report instruments. These models include behavioral indicators that can capture clinically relevant levels of impairment. For example, the models are not concerned with whether a patient walked 9000 steps versus 10,000 steps, but if the patient has not left his house in the past week. The models use macro level behavioral features as inputs, such as distance traveled over a week, to predict symptoms that both patients and clinicians can understand and potentially intervene upon. The present models do not assume a causal link between the behavioral indicators and clinical symptoms; nevertheless, the models may be of interest to clinicians and patients regardless of their causal pathways, as a means to track and alert to changes in symptoms in real time. These models are not designed to replace clinical decision-making or diagnose a particular mental health condition, but rather to assess a number of behavioral indicators underlying mood and anxiety disorders. These models are a resource to provide clinically relevant behavioral information to augment clinical care.

This study also demonstrates the feasibility and scalability of mobile sensing platforms for capturing, sharing, and analyzing behavioral data. Participants reported comfort with sharing even personal data with clinicians and medical researchers. They were willing to use the app in the future and reported that it did not change their behavior. Future research should continue to explore feasibility and acceptability of mobile sensing platforms in different populations and how this technology influences patients, clinicians, and their interactions.

### Clinical Relevance

The behavioral indicator algorithms created in this study, when embedded within a high performance, highly scalable, cloud computing architecture has distinct advantages in clinical care. The models described in this study utilize only 1 week of data to make predictions, and when implemented in high-performance code can automatically provide new values on patient behavior daily. Continuous monitoring can allow patients, clinicians, or researchers to view changes and symptom trajectories over time. Passive data collection does not burden patients and is free from self-report subject to biases. Current mHealth approaches for mental health assessment, including mobile phone apps and ecological momentary assessment methods, may be an improvement over paper and pencil measures, but still require direct patient input [22,33] and many have not been clinically validated [34]. The use of mobile sensing platforms could allow clinicians and researchers to track episode onset, symptom progression, and relapse across populations with less patient burden [35]. Patients, caregivers, or clinicians could be alerted to clinically meaningful changes in behavioral indicators between clinical interactions.

Currently, there is no real-time, continuous, and objective solution to collect, analyze, and track mental health symptom-related behavioral indicators. The mobile sensing approach validated in this study streamlined the process by which clinically-relevant indicators are gathered and transmitted. Each year, approximately 6.9% of all US adults experience major depressive disorder [36] and PTSD prevalence is estimated at 6.8% over a lifetime [37]. The rates of mood and anxiety disorders are even higher among veterans. Although estimates vary by study and service era, current prevalence of depression in veterans of Iraq and Afghanistan conflicts is estimated at 13.8% [38] and lifetime prevalence of PTSD is estimated at 30.9% for male veterans and 26.9% for female veterans [39]. This innovative mobile sensing platform offers a scalable approach to care for millions with mood and anxiety disorders.

This study sample is larger than in previous studies of mobile sensing platforms and weighted to prevalence estimates; however, it is a convenience sample from the community and, subsequently, results may lack generalizability. Although the study demonstrates proof of concept, additional validation of these models is needed.

The use of this innovative approach could empower patient health self-management. Recognizing warning signs and changes in behavioral patterns can be difficult for patients with mood and anxiety disorders, particularly those without easy access to care or those experiencing symptoms near episode onset. Disability and behavior are closely linked and mental disorders represent a leading cause of disability. Developing approaches to identify functional disability that do not rely on self-report is an important extension to this work. Patients who are more aware of their own behavioral patterns, particularly between or before clinical appointments, maybe are better able to manage their own health. A mobile sensing platform could be capable of providing real-time feedback to patients and clinicians, enabling increased help seeking behavior and access to needed care. Patients and providers can use behavioral data to recognize more objectively and definitively when they need to seek additional services. These additional novel metrics may enable clinicians to provide better informed clinical care. The efficacy of such a platform in improving clinical care remains to be tested.

## Appendix

Multimedia Appendix 1 Supplemental material: eMethods: Participants - Mobile sensing platform architecture - Data security & privacy - Digital trace data - Survey instrument; eTables: Features Tables - Audio Feature Definitions - Candidate Models - Participant Characteristics - Detailed model performance.

## Abbreviations

AUC: area under the curve
GPS: global positioning system
LASSO: least absolute shrinkage and selection operator
PTSD: post-traumatic stress disorder
RDoC: Research Domain Criteria
ROC: receiver operating characteristic
SCID: Structured Clinical Interview for Mental Disorders
