# Supplementary material for: Behavioral Indicators on a Mobile Sensing Platform Predict Clinically Validated Psychiatric Symptoms of Mood and Anxiety Disorders
Source: J Med Internet Res. 2017 Mar 16;19(3):e75. doi: 10.2196/jmir.6678 (PMC5374272; doi:10.2196/jmir.6678)
Supplement: Multimedia Appendix 1 [file jmir_v19i3e75_app1.pdf]

SUPPLEMENTARY ONLINE CONTENT

Place et al:
Behavioral Indicators on a Mobile Sensing Platform Predict Clinically Validated Psychiatric Symptoms of Mood and Anxiety Disorders

This supplementary material has been provided by the authors to give readers additional information about their work.

Contents:

eMethods – Participants
eMethods – Mobile sensing platform architecture
eMethods – Data security & privacy
eMethods – Digital trace data
eMethods – Survey instrument

eTable 1. Features Tables
eTable 2. Audio Feature Definitions
eTable 3. Candidate Models
eTable 4. Participant Characteristics
eTable 5. Detailed model performance

eMethods - Participants

During an additional in-person screening before consent, participants filled out the Beck Scale for Suicide Ideation (BSS; Beck et al., 1993). If participants had a positive response on any item, they met with a clinical social worker who administered the Mini-International Neuropsychiatric Interview (M.I.N.I.) suicide module and excluded participants at immediate risk of self-harm or self-reported suicidality. No participants were excluded for this reason.

eMethods – Mobile sensing platform architecture

Cogito Corporation developed a mobile sensing platform for use in data gathering, storage, and analysis. The software allows for data collection, secure transfer, ecological momentary assessment, and audio recording. Digital trace data was collected from predefined, configured probes built into the phone's operating system. Data from probes were gathered on intermittent fixed schedules. Database files were encrypted on the mobile device before being securely transferred to centralized servers. On the server, data was decrypted and then validated to assure data structural integrity. This step was critical as the underlying mobile operating system can update at any time and change the structure of the data files. Validated data were then stored in databases and encrypted at rest. These data are later transformed into feature and modeling results by the computational engine embedded within the platform software. The platform also includes an ecological momentary assessment system with the ability to present, collect, and store self-reported survey results from individuals. An audio recording functionality allows users to record audio check-ins to gather voice data.

eMethods – Data security & privacy

Protecting Identity

All data gathered and stored on the system were marked by a unique identification number (UID). The identities of the individuals with whom participants communicated were also protected, using a salted hash approach where a random string represented the same phone number per participant's social contact throughout the study. While a single hash was generated per social contact on each device, a different hash was created for the same contact on another individual's device. Thus, the salted hash method protected the identities of those in participants' social network, and, by extension, the identities of subjects themselves.

Data Storage on the Device

Once data were gathered on the participant's device, they were immediately encrypted, according to military grade AES, in a public-private key pairing. As the data were always stored in an encrypted format on the subject's device, the research application and any other app, as well as the user him or herself, could not access the data. Given the encrypted nature of the stored data, if a participant lost his or her mobile phone, there would have been an extremely minimal risk of privacy breach.

Data Storage on the Platform

Data were transferred from the participant's phone to Cogito servers through a Secure Sockets Layer (SSL), an encrypted connection. Once data reached Cogito servers, the information was decrypted for analysis, and encrypted at rest. All data were stored locally at Cogito, and identified by unique identification number only. Data access was limited to named study staff.

eMethods – Digital trace data

The mobile sensing platform collected six main categories of digital trace data over the 12-week study period: Activity, Social, Location, Device Interaction, Device Information, and Vocal Cues.

Activity data included Accelerometer and Gyroscope probes, both of which were gathered every 30 minutes. The Accelerometer variables were represented by the rate of rotation of the phone around the X, Y, and Z axes. The average amount of Accelerometer data gathered per week for each participant was 54,889 readings, and the total amount of Accelerometer data collected over the 12-week study period across all participants was 47,006,966 readings.

Social data was captured through Contacts, Call Log, and SMS Log probes. Data from the Contacts probe were gathered after every phone re-start while Call Log and SMS Log data were collected once each day. The average amount of Call and SMS Log data for each participant per week was 107 calls and 190 SMS messages. The total amount of gathered Call and SMS Log data was 90,976 calls and 166,948 SMS messages.

Location data was measured via a Location probe, from which data were gathered every 30 minutes. The average amount of Location data gathered per week for each participant was 250 GPS readings, and the total amount of data collected was 218,822 GPS readings.

Device Interaction was assessed through a Screen probe, which collected data every time the the phone screen was turned on/activated and turned off. The average amount of data gathered per week for each participant was 1,179 screen readings, and the total amount of data collected was 1,033,121 screen readings.

Device Information data were measured through the probes Battery, Android Info, and Hardware. Hardware and Android Info data were gathered once a day, while Battery data were collected every hour.

Vocal cues were sampled through participants' audio diaries. The average amount of data collected per week for each subject was approximately 1 audio diary, and the total amount of data gathered was 847 audio diaries.

eMethods – Survey instrument

Participants were asked if they felt the application violated personal privacy, if they consciously changed cell phone use because of study participation, if they would be interested in using a similar application in the future, and if they would like to receive personalized feedback. They were also asked if they would be willing to share personalized health information collected through the application with friends, family, healthcare providers, mental health providers, similar patients, researchers, insurance providers, or government health organizations. These were averaged to create a comfort with personal sharing score. Participants were also asked if they would be willing to share anonymized health-related data with researchers, government organizations, other patients, support groups, and insurance providers. These were averaged to create a comfort with anonymize sharing score. All responses were on a 5-pt scale from not at all to extremely.

In the survey below, we refer to the application as Cogito VetGuard.

Q. How easy was it to answer survey questions on your phone?

1	2	3	4	5
Not easy At all				Extremely easy

Additional comments (optional):

Q. How easy was it to leave audio diaries on your phone?

1	2	3	4	5
Not easy At all				Extremely easy

Additional comments (optional):

Q. How much did you consciously change your behavior because of the application on your phone?

1	2	3	4
None	A little	Some	A lot

Q. Did any of the following change because of the application on your phone?

The places I visited	Yes	No
The people I spent time with	Yes	No
My behavior at home	Yes	No
My behavior at work	Yes	No
My behavior in other social situations	Yes	No

Q. How much did you consciously change your cell phone use because of the application on your phone?

1	2	3	4
None	A little	Some	A lot

Q. Did any of the following change because of the application on your phone?

How often I carried my phone with me	Yes	No
How often I used my phone	Yes	No
How OFTEN I texted	Yes	No
How OFTEN I made phone calls	Yes	No
WHO I texted	Yes	No

WHO I called	Yes	No
How often I downloaded other apps	Yes	No
What other apps I downloaded	Yes	No
What other apps I used	Yes	No
How often I took pictures with my phone	Yes	No
What I took pictures of with my phone	Yes	No

Q. I felt that the application on my phone violated my personal privacy.

1	2	3	4	5
Not at all	A little bit	Moderately	Quite a bit	Extremely

In the future, a mobile phone application, like Cogito VetGuard, might be able to provide you with information about your mental health.

Would you be interested in using such an app?

1	2	3	4	5
Not at all	A little bit	Moderately	Quite a bit	Extremely

If you used this future mobile phone app, would you share the data from this future app with your:

	Not at all (1)	A little bit (2)	Moderately (3)	Quite a bit (4)	Extremely (5)
Principal care provider or mental health specialist?					
Family or friends?					
Veterans with whom you served					
With other anonymous veterans?					

Q. How likely would you be to download an audio diary service mobile application if it provided feedback on your mental health?

1	2	3	4	5
Not at all	A little bit	Moderately	Quite a bit	Extremely

What did you find to be the most helpful part of this study?

What did you find to be the least helpful part of this study?

What suggestions do you have to improve the Cogito VetGuard application?

On a scale of 1 to 5, to what degree do you feel participation in our study helped you? (Circle the number corresponding to your response)

1	2	3	4	5
Didn't help				Helped me a lot

How helpful was the Cogito VetGuard application in improving your quality of life?

1	2	3	4	5
Didn't help				Helped me a lot

How did it help you? or Why didn't it help you? (write comments below)

If you could, what would you change about the application?

eTable 1. Features Tables

Social Data – Call Log Probe

Digital Trace Data Category	Probe	Feature Name	Feature Definition
Social	Call Log	Call.number.count	Count of the phone numbers in calls
Social	Call Log	Call.out.sum	Count of calls originated
Social	Call Log	Call.in.sum	Count of calls received
Social	Call Log	Call.missed.sum	Count of calls missed
Social	Call Log	Call.duration.sum	Total duration of calls
Social	Call Log	Call.out.mean	Fraction of all calls that were originated
Social	Call Log	Call.in.mean	Fraction of all calls that were received
Social	Call Log	Call.missed.mean	Fraction of all calls that were missed
Social	Call Log	Call.duration.mean	Mean duration of all calls

Social Data – SMS Log Probe

Digital Trace Data Category	Probe	Feature Name	Feature Definition
Social	SMS Log	Sms.address.count	Count of the phone addresses in SMS
Social	SMS Log	Sms.out.sum	Count of SMS originated
Social	SMS Log	Sms.in.sum	Count of SMS received
Social	SMS Log	Sms.elapsed.sum	Total elapsed time between SMS
Social	SMS Log	Sms.out.mean	Fraction of all SMS that were originated
Social	SMS Log	Sms.in.mean	Fraction of SMS that were received
Social	SMS Log	Sms.elapsed.mean	Mean elapsed time between SMS

Location Data – Location Probe

Digital Trace Data Category	Probe	Feature Name	Feature Definition
Location	Location	Travel.distance.sum	Total distance traveled; calculated by taking the latitude/longitude every 30 minutes and summing distances traveled in a week period, where distances were computed using the great circle distance in meters between 2 sequential GPS locations.
Location	Location	Travel.elapsed.sum	Total time elapsed during travel
Location	Location	Travel.variance.sum	Variance of total distance traveled

*Vocal Cues – Audio Probe**

Digital Trace Data Category	Probe	Feature Name	Feature Definition
Vocal Cues	Audio	mean_EPDC	Mean speaking fraction
Vocal Cues	Audio	mean_EPDC2	Mean speaking rate
Vocal Cues	Audio	mean_Harm	Mean Harmonicity
Vocal Cues	Audio	sd_Harm	Standard deviation of Harmonicity
Vocal Cues	Audio	mean_VE0	Mean vocal effort feature-1
Vocal Cues	Audio	sd_VE0	Standard deviation of vocal effort feature-1
Vocal Cues	Audio	mean_PV	Mean pitch variation feature-1
Vocal Cues	Audio	sd_PV	Standard deviation of pitch variation feature-1
Vocal Cues	Audio	mean_VE1	Mean vocal effort feature-2
Vocal Cues	Audio	sd_VE1	Standard deviation of vocal effort feature-2
Vocal Cues	Audio	mean_PV2	Mean pitch variation feature-2
Vocal Cues	Audio	sd_PV2	Standard deviation of pitch variation feature-2
Vocal Cues	Audio	mean_PV3	Mean pitch variation feature-3
Vocal Cues	Audio	sd_PV3	Standard deviation of pitch variation feature-3

*For a more detailed explanation of feature definitions, please refer to the table below

eTable 2. Audio Feature Definitions

Time level feature	Description	Reference	Audio-diary level feature statistics
Speaking Fraction	Proportion of audio diary recording with detected speech activity	Speech activity detection based voicing detection algorithm described in: Boersma, P 1993. ‘‘Accurate short-term analysis of the fundamental frequency and the harmonics-to-noise ratio of a sampled sound,’’ Proc. Institute of Phonetic Sciences 17, 97–110	Mean speaking fraction (mean_EPDC)
Speaking Rate	Pace of subject speaking	Morgan, Nelson, and Eric Fosler-Lussier. "Combining multiple estimators of speaking rate." Acoustics, Speech and Signal Processing, 1998. Proceedings of the 1998 IEEE International Conference on. Vol. 2. IEEE, 1998.	Mean speaking rate (mean_EPDC2)
Harmonicity	A measure of the strength of harmonicity or periodicity in the audio signal. Here we consider harmonicity only in voiced speech regions, which tend to be highly periodic (due to regular vocal fold vibration). However, certain variations of phonation type involving less regular vocal fold vibrations (e.g., breathy, harsh or creaky voices) will have lower harmonicity values.	Harmonicity is computed as part of our implementation of Paul Boersma's pitch tracking algorithm: Boersma, Paul. "Accurate short-term analysis of the fundamental frequency and the harmonics-to-noise ratio of a sampled sound." Proceedings of the institute of phonetic sciences. Vol. 17. No. 1193. 1993.	Mean Harmonicity (mean_Harm)
			Standard deviation Harmonicity (sd_Harm)
Vocal Effort	Feature characterizing breathy and tense phonation types	Statistical perceptual model based on variations of the features described in: M. Lugger, B. Yang, W. Wokurek, Robust estimation of voice quality parameters under real-world disturbances, in: 2006 IEEE International Conference on Acoustics, Speech and Signal Processing, 2006, ICASSP 2006 and Hillenbrand,J., and Houde, R. A. (1996). ”Acoustic Correlates of Breathy Vocal Quality: Dysphonic Voices and Continuous Speech”, J. Speech Hear. Res. 39, 311–321.	Mean Vocal Effort-1 (mean_VE0)
			Mean Vocal Effort-2 (mean_VE1)
	Feature variants: Vocal Effort-1, Vocal Effort-2		Standard deviation of Vocal Effort-1 (sd_VE0)
			Standard deviation of Vocal Effort-2 (sd_VE1)
Pitch Variation	Feature characterizing monotonous to highly varied pitch patterns.	Feature based on an extension of the algorithm described in K. Laskowski, J. Edlund, and M. Heldner, “An instantaneous vector representation of delta pitch for speaker-change prediction in conversational dialogue systems,” in Proc. ICASSP, Las Vegas NV, USA, 2008, pp. 5041–5044.	Mean Pitch-Variation-1 (mean_PV)
			Mean Pitch-Variation-2 (mean_PV2)
			Mean Pitch-Variation-3 (mean_PV3)
	Feature variants: Pitch variation-1, Pitch variation-2, Pitch variation-3		Standard deviation Pitch-Variation-1 (sd_PV)

			Standard deviation Pitch-Variation-2 (sd_PV2)
			Standard deviation Pitch-Variation-3 (sd_PV3)

eTable 3. Candidate Models

**Candidate Model Definition****
sms.address.count + travel.distance.sum + call.out.sum
call.out.sum + sms.address.count
call.out.sum + sms.address.count + call.out.sum*sms.address.count
sms.address.count + travel.distance.sum
sms.address.count + travel.distance.sum + sms.address.count *travel.distance.sum
call.out.sum + travel.distance.sum
call.out.sum + travel.distance.sum + call.out.sum*travel.distance.sum
A1 ~ mean_Speaking + mean_VE0 + mean_PV + mean_PV3 + sd_VE1
A1 ~ sd_VE0 + mean_VE1 + sd_PV
A1 ~ sd_VE0 + mean_VE1 + mean_PV
A1 ~ mean_VE1 + mean_PV
A1 ~ mean_VE1 + mean_PV + mean_VE1:mean_PV
A1 ~ mean_VE1 + mean_PV + mean_Speaking

**All four targets used the same candidate models

RESULTS

eTable 4. Participant Characteristics

Gender	
Male	49
Female	24
Age	
18-30	23
30-40	20
40-50	20
50-60	9
60-70	1
Education	
Some high school	2
High school/GED	14
Some college	27
Associate degree	10
Bachelor degree	15
Graduate/advanced degree	5
Race/Ethnicity	
White (Non-Hispanic)	35
Hispanic/Latino(a)	7
NH African American	23
NH Other/Not reported	8
Marital Status	
Married	14
Divorced/Separated/Widowed	17
Single	42
Veteran Status	
Yes	26
No	46
Missing value	1
Combat Experience	
Yes	9
No	17
Family HX of Mental illness	
Yes	42
No	31
History of Suicidality	
Yes	14
No	59

eTable 5. Detailed model performance

Symptom			Model	Evaluations cross validated (10-folds 100 runs mean and sd)													
Symptom	Description	Mental	Model	model	threshold	Accuracy		Sensitivity		Specificity		PPV (0. 5		NPV (0.5		auc	
						mean	sd	mean	sd	mean	sd	mean	sd	mean	sd	mean	sd
A1	Depressed mood most of the day	Depression	MeanPitchVar + MeanVocalEffort+ MeanVocalEffort:MeanPitchVar	A1	-											0.74	0.034
				A1	0.1	0.6704412	0.0342242	0.788	0.0624095	0.602093	0.0425741	0.6644196	0.0288057	0.7434097	0.0584552		
				A1	0.2	0.6725	0.0274808	0.608	0.0505325	0.71	0.0370231	0.67729	0.032121	0.6452578	0.0292582		
				A1	0.3	0.7007353	0.0306038	0.548	0.0438547	0.7895349	0.0395791	0.7234606	0.0421089	0.6363295	0.0259312		
				A1	0.4	0.6954412	0.0300942	0.4452	0.0494511	0.8409302	0.0401743	0.738531	0.0522874	0.6029363	0.02357		
				A1	0.5	0.6967647	0.0336243	0.3788	0.0503579	0.8816279	0.0432362	0.7647013	0.0702768	0.5869449	0.0234653		
				A1	0.6	0.6957353	0.0323607	0.3228	0.0518755	0.9125581	0.0369736	0.7883796	0.0798094	0.5743719	0.0223913		
				A1	0.7	0.6913235	0.0268894	0.2624	0.048682	0.9406977	0.0317692	0.8188104	0.0809662	0.5608115	0.0182974		
				A1	0.8	0.6801471	0.0263046	0.2188	0.0528631	0.9483721	0.0279656	0.8099352	0.0874721	0.5487181	0.018458		
				A1	0.9	0.6705882	0.0229922	0.1788	0.040384	0.9565116	0.0257432	0.8069754	0.0957305	0.5382348	0.0145869		
A2	Diminished interest or pleasure in all or most activities	Depression	sms.address.count + travel.distance.sum	A2	-											0.562277	0.0237276
				A2	0.1	0.5754167	0.0252617	0.5594118	0.0446306	0.5841935	0.0342785	0.5734356	0.0267429	0.570648	0.0260805		
				A2	0.2	0.6535417	0.0289608	0.3923529	0.0508946	0.7967742	0.0373892	0.658455	0.0517925	0.5677178	0.0216389		
				A2	0.3	0.7127083	0.028293	0.3594118	0.0602298	0.9064516	0.0249027	0.7913915	0.0579719	0.5866585	0.0239555		
				A2	0.4	0.6852083	0.0256261	0.2270588	0.0597198	0.9364516	0.0168181	0.7713459	0.0816301	0.5484276	0.020218		
				A2	0.5	0.6770833	0.0186104	0.1494118	0.0421538	0.9664516	0.0151512	0.8042496	0.1396283	0.5321272	0.0130238		
				A2	0.6	0.665625	0.0190469	0.1082353	0.0448065	0.9712903	0.0136744	0.7526498	0.2189232	0.5216128	0.0132535		
				A2	0.7	0.6458333	0.0172662	0.0247059	0.0303533	0.9864516	0.0172653	NaN	0.4650182	0.5029254	0.0098885		
				A2	0.8	0.646875	0.0061936	0.0041176	0.0150843	0.9993548	0.0045389	NaN	0.4409586	0.5008967	0.004096		
				A2	0.9	0.6458333	0	0	0	1	0	NaN	NaN	0.5	0		
A6	Fatigue or loss of energy	Depression	call.out.sum + sms.address.count	A6	-											0.7525	0.0520123
				A6	0.1	0.7708	0.0358921	0.8657895	0.0405446	0.47	0.0685754	0.621366	0.0317293	0.777933	0.0639714		
				A6	0.2	0.7168	0.0344328	0.78	0.0419054	0.5166667	0.0603953	0.6185381	0.033907	0.7018485	0.0466273		
				A6	0.3	0.6884	0.0351826	0.7357895	0.0395701	0.5383333	0.0780479	0.6167921	0.0441128	0.669481	0.0453402		
				A6	0.4	0.6972	0.0455733	0.7094737	0.0541927	0.6583333	0.0958599	0.6796746	0.0632873	0.6928634	0.0489071		
				A6	0.5	0.6576	0.04033	0.6178947	0.047087	0.7833333	0.0932636	0.747621	0.0709989	0.6709085	0.0390777		
				A6	0.6	0.6496	0.0386102	0.6010526	0.0449964	0.8033333	0.0725517	0.75782	0.0590133	0.6676529	0.0346218		
				A6	0.7	0.6208	0.0411864	0.5631579	0.0476071	0.8033333	0.0725517	0.7456041	0.0620387	0.6472552	0.0359948		
				A6	0.8	0.574	0.0399495	0.5015789	0.0469889	0.8033333	0.0725517	0.7233784	0.063985	0.6165391	0.0338662		
				A6	0.9	0.5452	0.0384151	0.4621053	0.0458587	0.8083333	0.0763028	0.7138106	0.0749517	0.5997315	0.0321583		
C2	Avoid activities, places, people	PTSD	call.out.sum + sms.address.count + (call.out.sum)(sms.address.count)	C2	-											0.8306597	0.0429699
				C2	0.1	0.7772	0.0448585	0.775625	0.0671238	0.78	0.0473253	0.7799136	0.0398725	0.7799136	0.0507628		
				C2	0.2	0.7816	0.0537619	0.7425	0.0800883	0.8511111	0.0552065	0.8348106	0.0507789	0.8348106	0.0553278		
				C2	0.3	0.7324	0.0484741	0.648125	0.0746705	0.8822222	0.0308664	0.8459573	0.0321434	0.8459573	0.043892		
				C2	0.4	0.6756	0.0447286	0.559375	0.0684509	0.8822222	0.0308664	0.8258003	0.0349773	0.8258003	0.0358933		
				C2	0.5	0.6604	0.0434827	0.535	0.0654723	0.8833333	0.0243381	0.8200625	0.0336284	0.8200625	0.0338001		
				C2	0.6	0.6544	0.0423626	0.525625	0.0628745	0.8833333	0.0243381	0.8174774	0.0346229	0.8174774	0.0328096		
				C2	0.7	0.6444	0.041738	0.509375	0.06305	0.8844444	0.0218829	0.8140391	0.0319125	0.8140391	0.0317608		
				C2	0.8	0.6264	0.035605	0.47375	0.0519354	0.8977778	0.0341647	0.8243565	0.0588916	0.8243565	0.0252771		
				C2	0.9	0.6512	0.0406334	0.469375	0.0514123	0.9744444	0.0495773	0.9548219	0.0867739	0.9548219	0.0280283		
